# Strength-Cognitive Training: A Systemic Review in Adults and Older Adults, and Guidelines to Promote “Strength Exergaming” Innovations

**DOI:** 10.3389/fpsyg.2022.855703

**Published:** 2022-05-27

**Authors:** Samad Esmaeilzadeh, Susanne Kumpulainen, Arto J. Pesola

**Affiliations:** Active Life Lab, South-Eastern Finland University of Applied Sciences, Mikkeli, Finland

**Keywords:** cognitive function, dual-task, exergaming, function, strength training

## Abstract

**Background:**

Despite functional and cognitive benefits, few adults and older adults do strength training twice per week with sufficient intensity. Exercise-based active video games (exergaming) may amplify the cognitive benefits of exercise and increase adherence and motivation toward training. However, the benefits of a well-defined and monitored dose of strength training, executed simultaneously or sequentially with a cognitive element, has received little attention. In this study we have two aims: First, to systematically gather the available evidence; second, to suggest possible ways to promote strength exergaming innovations.

**Methods:**

We systematically reviewed randomized controlled trials using simultaneous or sequent combined strength and cognitive training or strength exergaming to improve cognitive or functional outcomes in adults and older adults.

**Results:**

After screening 1,785 studies (Google Scholar, ACM Digital Library, IEEE Xplore Library, PsycARTICLES, Scopus, Cochrane Library and PubMed) we found three eligible studies. Of the two studies using sequent strength and cognitive training, one showed improved functionality, but the other showed negative effects on cognition. The third study using simultaneous intervention, reported a positive influence on both cognition and function, when compared with either strength training alone or a control group. Moderate level of evidence was showed on GRADE analysis.

**Conclusion:**

The existing little evidence suggests that strength and cognitive training improves cognition and function in adults and older adults. The following suggestions may help to promote further innovation: (1) ensure minimal dosage of strength training (30–60 min, 2 × /week), (2) use machine-based strength training devices to control volume and intensity (to prevent cognitive components from interfering with strength training), (3) include power training by using cognitive tasks requiring rapid reactions, and (4) add cognitive memory tasks (to extend the cognitive benefits of strength training *per se*), and (5) include motivational exergame elements to increase adherence.

## Highlights

– Strength training has significant benefits for adults and especially for older adults.
– Few adults and older adults do the recommended amount of strength training.
– Exergaming amplifies the cognitive benefits of exercise and increases adherence and motivation toward training.
– There are a few studies using strength cognitive training interventions and their results showing positive effects on either cognition or function.
– We provided some guidelines which may help to promote strength exergaming innovations.

## Introduction

Aging is a progressive and dynamic process with functional, morphological, psychological, and biochemical changes, many of which may lead to health challenges or difficulties (Cummings and Kropf, 2011; Kaeberlein et al., 2015). Functional deficiency, chronic diseases, neurodegenerative diseases, and other age-related problems are an increasingly important global challenge given increased life expectancy and number of older adults (Kontis et al., 2017). Despite progression in medicine, social care, and health care, increased life expectancy does not directly commensurate with increased health (St Sauver et al., 2015). Participation in physical exercise brings numerous health benefits and slows the negative health-related effects of aging (Gremeaux et al., 2012). Numerous exercise interventions in either healthy or unhealthy older adults have been performed to find the most effective counter-measures for aging-related challenges. In their recent meta-analysis and review, Di Lorito et al. (2021) reported that strength training, exercise-based active video games (exergaming), and meditative movement are the most effective exercise interventions to improve health outcomes such as cognition and function in older adults.

Given that exergaming is typically performed with aerobic type of activities (Stojan and Voelcker-Rehage, 2019; Gallou-Guyot et al., 2020), it remains unknown if combining strength training with exergaming could further improve the health of adults, especially the older adults.

Strength training increases both muscle mass and strength, which is one of the main mechanisms that link strength training to several health benefits. Muscle weakness has been linked with a variety of age-related negative outcomes such as cognitive decline (Boyle et al., 2009; Fragala et al., 2019; Herold et al., 2019), diabetes (Peterson et al., 2016), osteoporosis (McGrath et al., 2017), and early all-cause mortality (McLean et al., 2014). Strength training not only prevents or even reverses the decrease of muscle mass and strength (Fragala et al., 2019), but it also decreases intramuscular adiposity (Goodpaster et al., 2008), improves metabolic health and insulin sensitivity (McLeod et al., 2019), blood pressure, gastrointestinal transit time (Winett and Carpinelli, 2001), muscle quality (Evans, 2002; Goodpaster et al., 2008), bone density (Marques et al., 2012), physical performance (Häkkinen et al., 2002; Fragala et al., 2019), sarcopenia and lower-back pain (Winett and Carpinelli, 2001), psychological well-being (Fisher et al., 2017), as well reduces the risk for falls (Silva et al., 2013) and postpone disability and independent living (Spirduso and Cronin, 2001). The benefits of strength training in decreasing the risk of various chronic diseases such as diabetes, mobility, disability, cardiovascular diseases and cancer in older adults are similar to those of aerobic training (Westcott, 2012; Shaw et al., 2015; McLeod et al., 2019). In addition, strength training provides an effective alternative to aerobic training for older adults who are physically limited or have cardiorespiratory problems such as asthma, and thus are not able to participate in aerobic exercise training such as cycling or jogging (Ouellette et al., 2004; Yerokhin et al., 2012).

Recent meta-analysis and review studies concluded that strength training benefits functional brain changes and increases cognitive function in both healthy or cognitively impaired adults and older adults (Li et al., 2018; Herold et al., 2019; Landrigan et al., 2020; Huang et al., 2021). Most recently, Daniel Gallardo-Gomez et al. (2022) in a review and meta-analysis study suggested superior impact of strength training on cognition compared to other modalities such as aerobic exercise in older adults (Daniel Gallardo-Gomez et al., 2022). These benefits happen independent of increased cardiorespiratory fitness (Mavros et al., 2017). Despite the relatively small number of studies available and the highly variable results (Landrigan et al., 2020), there are many plausible potential mechanisms support that these benefits. Changes in hormone levels (Kraemer and Ratamess, 2005), and increases in cerebral blood flow (Timinkul et al., 2008), proteins such as insulin-like growth factor 1 (IGF-1) (Cotman et al., 2007), as well as brain-derived neurotropic factor (BDNF) (Bramham and Messaoudi, 2005), are some of the suggested mechanistic pathways linking strength training with cognitive and cerebral health benefits. A further possible mechanism may be that performing regular resistance exercise could function as a type of cognitive training (Landrigan et al., 2020).

To gain the established benefits of strength training (Westcott, 2012; Shaw et al., 2015; Fisher et al., 2017; Fragala et al., 2019; McLeod et al., 2019), older adults are recommended to engage in mild-to high-intensity workouts/trainings, with ≤ 60-min duration, twice a week (Nelson et al., 2007; Fisher et al., 2017; Fragala et al., 2019).

However, the vast majority of older adults do not engage in regular strength training (National Center for Health Statistics Survey, 2016). Of those who do, not all exercise at the recommended intensity or frequency (Gordon-Larsen et al., 2004; Cavill and Foster, 2018) and adherence to resistance exercise programs remains low (Gordon-Larsen et al., 2004; Nyman and Victor, 2012; Burton et al., 2017; Cavill and Foster, 2018). With age, individuals may face many barriers for participation, such as depression, risk of falling, loss of independence, cost, health concerns, safety concerns, pain, inaccessibility, fear, fatigue, and lack of motivation and social support (Gordon-Larsen et al., 2004; Burton et al., 2017; Cavill and Foster, 2018). Therefore, there is a need to develop motivating and engaging, yet safe and effective, strength training regimens to increase strength training participation rates in adults and especially older adults because of the direct functional benefits they can gain.

Exergaming is a unique form of dual-task training in which cognitively challenging tasks are combined with physical exercise with an interactive game-based method (Stojan and Voelcker-Rehage, 2019; Gallou-Guyot et al., 2020). The main difference between dual-task training and exergaming is that in exergame an individual has to complete the cognitively challenging task while gaming physically. However, in dual-task training there is a distinct training task (e.g., counting forward/backward while walking) without the gaming element (Stojan and Voelcker-Rehage, 2019; Gallou-Guyot et al., 2020). It is believed that the playful character of exergaming encourages and motivates older adults in physical exercise participation and improves adherence (Skjæret et al., 2016; Stojan and Voelcker-Rehage, 2019; Gallou-Guyot et al., 2020). In addition, exergaming is one of the most effective interventions in improving various health outcomes (i.e., cognition, function, etc.) in older adults (Di Lorito et al., 2021). Thus, one possible and interesting solution to increase not only motivation but also the effectiveness of strength training on cognition and function (Skjæret et al., 2016; Stojan and Voelcker-Rehage, 2019; Gallou-Guyot et al., 2020) may be to combine strength training with simultaneous cognitive video games as “strength exergaming.”

This study has two aims: Firstly, to systematically review the available randomized controlled trials that used combined strength and cognition training (either sequent or simultaneous) to improve cognition and/or functional outcomes in ≥55-year-old adults. Secondly, to discuss factors that future innovations should consider to maximize the effectiveness of strength cognition training.

## Methods

### Research Process

The review process followed the PRISMA (Preferred Reporting Items for Systematic Reviews and Meta-Analyses) guidelines (Moher et al., 2016). The following databases were used to search and collect the articles published in peer-review journals: Google Scholar, ACM Digital Library, IEEE Xplore Library, PsycARTICLES, Scopus, Cochrane Library and PubMed. The last research was performed on March 03, 2022. To limit the study, some restrictions were made to choose only academic publications with full text in the English language, and included age, gender, or ethnicity without restrictions. The following syntax search strategy was used: (middle-aged OR adult OR aging OR old OR older OR elderly OR senior) AND (dual-task OR exergame OR virtual reality OR active video game) AND (executive OR cognition OR processing speed OR dual-task OR memory OR reaction time OR attention OR verbal) AND (strength OR gait analysis OR walking OR balance OR agility OR fall) AND (strength training OR resistance training OR resistance exercise OR strength exercise). The quality of the included methodologies was assessed by using the “risk of bias tool” (Higgins et al., 2011), and the Joanna Briggs Institute's (JBI) critical appraisal tool (Tufanaru et al., 2020). A cut off point of at least 60% of the question used to show the adequate quality (Tufanaru et al., 2020). In addition, to observe the quality of the evidence for the outcome (certainty in the estimates of effect) we used narrative Grading of Recommendations Assessment, Development and Evaluation (GRADE) approach for systemic review without meta-analysis (Murad et al., 2017). Using the narrative grade the quality of or certainty in, the body of evidence can be judged to 4 levels of high certainty, moderate certainty, low certainty and very low certainty based on the risk of bias, inconsistency, indirectness, imprecision, and publication bias (Murad et al., 2017).

### Eligibility Criteria

Due to the limited number of strength-cognition interventions targeting cognition and function in healthy adults and older adults (≥55 years of ages), we also decided to incorporate studies that included subjects with cognitive or neurodegenerative problems (i.e., mild cognitive impairment, multiple sclerosis, Parkinson, and Alzheimer's diseases). We included studies with sequent or simultaneous strength-cognitive (i.e., strength-based dual-task and strength-based exergame). Those studies that combined strength training with other physical training such as aerobic exercise training or motor training (i.e., walking, balance, coordination, dance, etc.) were not included in the study. We selected only studies with primary or secondary outcome measures from the following domains: 1- Cognitive function 2- Measures of cognitive state such as Montreal Cognitive Assessment, Mini-Mental State Examination 3- Dual-task measurements 4- Related data to brain functional or structural data such as electroencephalography (EEG), and 5- Measurements related to function such as balance, strength, sit and stand, walking ability tasks (e.g., gait analysis, timed up and go, walking time, etc.).

The title and abstract of the collected studies were first analyzed and non-eligible studies were removed. Next, the full text was read to identify the articles that would be included in the study. The screening of the studies was performed by two independent researchers and any inconsistencies were discussed with all authors together.

## Results

**Figure 1:**
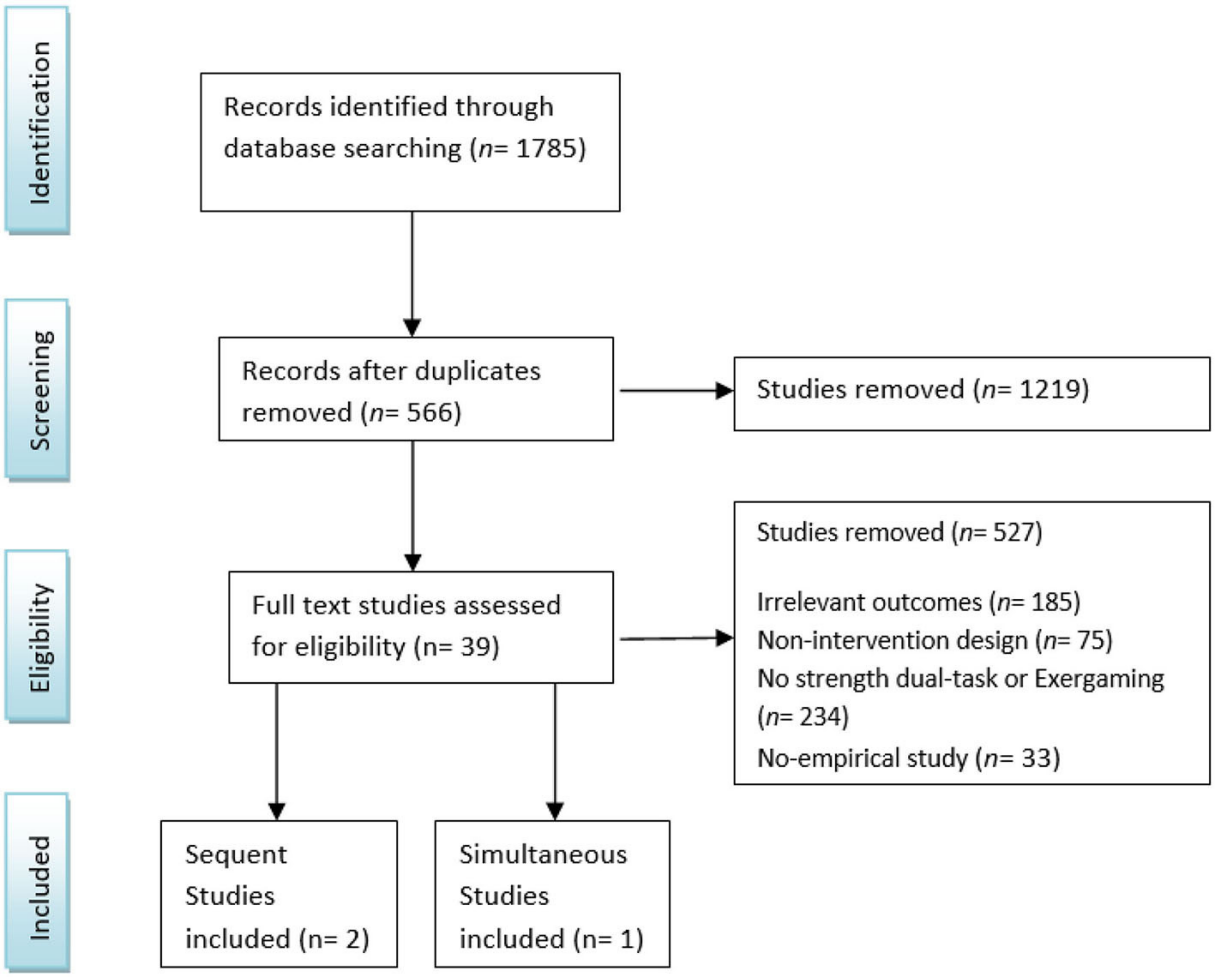
Studies selection PRISMA flow chart.

The details of the included studies are reported in Table 1. There were only one simultaneous and two sequent strength-cognitive based studies that met the eligibility criteria (Figure 1; Fiatarone Singh et al., 2014; Norouzi et al., 2019; Gutiérrez-Cruz et al., 2020). Only one study included healthy older adults (Norouzi et al., 2019) and in another study the age range of the subjects was 26–61 years (Gutiérrez-Cruz et al., 2020). However, due to the limited body of evidence we did not remove that study. None of the studies matching the search criteria used strength training based exergame. Two studies had a high risk of bias (Norouzi et al., 2019; Gutiérrez-Cruz et al., 2020) due to blinding and allocation concealment procedures. Furthermore, according to the JBI one study (Gutiérrez-Cruz et al., 2020) did not pass the quality criteria (Table 2). And finally, according to narrative grade moderate level of evidence was observed due to serious imprecision and borderline risk of bias (Tables 3, 4).

**Table 1:** Summary of the available studies examined the effects of sequent or simultaneous strength-cognitive interventions in adults or older adults.

**References**	**Participants**	***N***	**Sequent/** **Simultaneous**	**Study design**	**Duration (weeks)**	**Simultaneous intervention**			**Control group**	**Measures**	**StCT/** **StDT**	**St**	**CT**	**CON**	**Follow-up (weeks)**	**StCT/StDT**	**St**	**CT**	**CON**	**Risk of bias**
						**Cognitive**	**Exercise**	**Dose**												
Norouzi et al. (2019)	Healthy Male adults ≥65 years old	60	Combined	RCT StCT St CON	4	Memory	Isokinetic exercise device	60–80 min, 3/week	No treatment	Working memory Balance	Y Y	N N	- -	N N	12	Y Y	Y Y		N N	High
Gutiérrez-Cruz et al. (2020)	Males and females with multiple sclerosis, 26–61 years old	31	Sequent	RTC StDT CON	24	Dual-task	dynamic strength machines, elastic bands or manual resistance	60 min, 1/week	No treatment	Strength Gait Analysis Stabilo metry Sit-to-Stand	Y Y Y Y			N N N N						High
Fiatarone Singh et al. (2014)	Males and females with MCI, ≥55 years old	100	Sequent	RTC CT St StCT CON	42	Computer based cognitive training	Pneumatic resistance machines	60–100 min, 2–3/week	Sham exercise and cognitive	Global function EF Memory Function Speed/ Attention Functional status	N N N Y N	Y Y N Y N	N N N Y N	N N N N N	126	N N N Y N	Y Y N Y N	N N N Y N	N N N N N	Low

*RCT, Randomized controlled trial; StCT, Simultaneous strength and cognition training; St, Strength training; StDT, Strength-dual-task training; CT, Cognitive group; CON, Control group; EF, Executive function*.

**Table 2:** Assessment of methodological quality of the included studies using the JBI critical appraisal checklist for RCTs.

**References**	**Critical Appraisal of Randomized Controlled Study**													**Total (Yes)**
	**Q1**	**Q2**	**Q3**	**Q4**	**Q5**	**Q6**	**Q7**	**Q8**	**Q9**	**Q10**	**Q11**	**Q12**	**Q13**	
Fiatarone Singh et al. (2014)	Y	Y	Y	Y	Y	Y	N	Y	Y	Y	Y	Y	Y	12
Gutiérrez-Cruz et al. (2020)	Y	N	Y	N	N	N	N	N	Y	N	Y	Y	N	5
Norouzi et al. (2019)	Y	N	Y	N	N	N	N	Y	Y	Y	Y	Y	Y	8

*Yes (Y), No (N)*.

*Q1. Was true randomization used for the assignment of participants to treatment groups? Q2. Was allocation to treatment groups concealed? Q3. Were treatment groups similar at the baseline? Q4. Were participants blind to treatment assignment? Q5. Were those delivering treatment blind to treatment assignment? Q6. Were outcomes assessors blind to treatment assignment? Q7. Were treatment groups treated identically other than the intervention of interest? Q8. Was follow-up complete? and if not, were differences between groups in terms of their follow-up adequately described and analyzed? Q9. Were participants analyzed in the groups to which they were randomized? Q10. Were outcomes measured in the same way for treatment groups? Q11. Were outcomes measured in a reliable way? Q12. Was appropriate statistical analysis used? Q13. Was the trial design appropriate, and any deviations from the standard RCT design (individual randomization, parallel groups) accounted for in the conduct and analysis of the trial?*

**Table 3:** Rating the certainty in evidence.

**GRADE domain**	**Judgement**	**Concerns about certainty domains**
Methodological limitations of the studies	Two out of three trials had high risk of bias (Norouzi et al., 2019; Gutiérrez-Cruz et al., 2020) due to blinding and allocation concealment procedures, and one study had low risk of bias (Fiatarone Singh et al., 2014).	Borderline
Indirectness	The included participants comparators and intervention in the studies all provided direct evidence to the research question. Although we observed no serious indirectness in the studies but we found variability in the interventions and outcome measure.	Not serious
Imprecision	The total number of participants included in all the studies was 191. So, due to low number of participants results are concerning for imprecision (Guyatt et al., 2011).	Serious
Inconsistency	Of the two studies using sequent strength and cognitive training, one showed improved functionality (Gutiérrez-Cruz et al., 2020), but the other showed negative effects on cognition (Fiatarone Singh et al., 2014). The study using simultaneous strength and cognitive training reported a positive influence on both cognition and function (Norouzi et al., 2019).	Not serious, borderline
Publication bias	We did not suspect publication bias due to the reason that both positive and negative trials were published, and also the search for studies was comprehensive	Not suspected

**Table 4:** Summary of findings of narrative GRADE.

**Outcome**	**Effect**	**Number of participants (studies)**	Certainty in the evidence^*^
Cognition and function	Two out of three studies showed positive effect and one study showed mixed results.	*N* = 191 (3 randomized trials)	Moderate certainty ⊕⊕⊕O (Because of serious imprecision and also borderline risk of bias)

* *Commonly used symbols to describe certainty in evidence in evidence profiles: high certainty ⊕⊕⊕⊕, moderate certainty ⊕⊕⊕O, low certainty ⊕⊕OO and very low certainty ⊕OOO*.

### Sequent Strength-Cognitive Interventions

Fiatarone Singh et al. (2014) included 100 community-dwelling men and women aged ≥55 with mild cognitive impairment diagnosis and randomized them into four groups as follows: 1- Cognitive training targeting executive function, memory, attention, and speed of information processing (+Sham Exercise); 2- Progressive resistance training using Pneumatic resistance machines (+Sham Cognitive); 3- Combined resistance and cognitive training; 4- Control group who received both sham cognitive and sham exercise training. Each session lasted for 60–100 min and were performed on 2–3 days per week for a total of 6 months, with an 18-month follow-up. The outcomes included various measures of cognitive functioning tasks such as global cognitive function, executive function, memory function, speed/attention, and functional status. The authors observed that strength training alone improved measures of global and executive function, and speed/attention after 6 months of training, and the effects were maintained at the 18-month follow-up. Neither cognitive training nor combined, sequent, strength and cognitive training had beneficial effects on any of the cognitive measurements.

Gutiérrez-Cruz et al. (2020) included 31 subjects (26–61 years of old) with a confirmed diagnosis of multiple sclerosis and randomized them into 2 groups as follows: 1- Sequent strength and dual-task training; 2- Control. The 24-week intervention consisted of 3 sessions per week each lasting 60 min. The intervention group performed general dynamic strength training using their own body as well dynamic strength against resistance using machines, elastic bands or manual resistance. Various measures of function (i.e., static strength, gait analysis, Stabilometry and sit-to-stand) were used. The authors observed a significant improvement of strength, balance, daily activities such as walking or sitting-to-standing, as well the dual-task costs of step length and walking velocity after the strength-cognitive intervention.

### Simultaneous Strength-Cognitive Dual-Task Interventions

Norouzi et al. (2019) included 60 healthy male older adults (≥65 years) and randomized them into three groups as follows: 1- strength-cognitive dual-task; 2- strength, and; 3- control. The strength group used an isokinetic exercise device and the strength-cognitive dual-task group was requested to perform memory tasks while performing the same strength programs. Each session lasted for 60–80 min and were performed three times per week for a total of 4 weeks, and the effects were followed up at 12 weeks from baseline. The results indicated a positive impact on working memory and balance performance only in the strength and cognition dual-task group. The authors observed the same results at 12 weeks follow-up.

## Discussion

In this study, we systematically reviewed the existing evidence regarding combined strength and cognitive training (either sequent or simultaneous) in adults and older adults. Of the two studies using sequent strength and cognitive training, one showed improved functionality, but the other showed negative effects on cognition. The study using simultaneous strength and cognitive training reported a positive influence on both cognition and function, when compared with either strength training alone, or a control group. Based on the low number of studies and the available evidence on plausible mechanisms, we suggest that authors and innovators further explore the possibilities of combined strength and cognitive training. “Strength exergaming” can encourage adults and older adults to participate in strength training and also possibly increase the effectiveness of strength training on cognition and function.

### Sequent and Simultaneous Strength-Cognition Interventions

Overall, there were only three studies that used combined strength-cognition interventions. One study used simultaneous (Norouzi et al., 2019) and two studies used sequent (Fiatarone Singh et al., 2014; Gutiérrez-Cruz et al., 2020) interventions. The results were mixed, such that two of the studies reported a positive influence on cognition and function (Norouzi et al., 2019; Gutiérrez-Cruz et al., 2020) and one study reported no benefits of adding a cognitive component to strength exercise training to benefit cognition (Fiatarone Singh et al., 2014). There was no evidence of “strength exergaming” in the literature, i.e., there were no interventions that would have specifically designed the cognitive component as a gamified element. Gutiérrez-Cruz et al. (2020) reported a significant positive influence of a sequent strength and dual-task training on functional ability (i.e., strength, balance, and walking ability) in older adults. However, they did not include strength training group, nor a dual-task or active control group. This limits inference on the added benefits of combined strength and cognitive training, compared to strength training alone. Fiatarone Singh et al. (2014) reported that strength training could benefit cognitive function both at following the intervention and follow-up times. However, they reported that adding a further sequent cognitive component to strength training did not improve cognitive function in older adults with mild cognitive impairment. Additionally, they observed no influence of cognitive training alone on cognitive function. They suggested that adding a further sequent cognitive component to strength training may increase stress and/or decrease physical activities and therefore inhibit cognitive benefits. Further studies are required to investigate the best combination of sequencing and dosing of strength and cognitive training for increasing the benefits and minimizing the interference.

According to a recent systematic review, simultaneous cognitive and physical exercise training including aerobic, strength and balance components (i.e., dual-task) is more effective than sequent interventions (Tait et al., 2017). In dual-task programs, physical and cognitive exercises are combined in a simultaneous session. Many of these tasks have been reported to have a positive impact on either cognitive or cognitive/physical related variables such as physical and motor fitness, and risk of falling (Tait et al., 2017). A variety of dual-task training regimen, comprising of multicomponent physical, motor and cognitive training, have been reported to be beneficial for older adults (Tait et al., 2017). However, as evidenced by the recent and the present systematic reviews, there are few studies of simultaneous strength and cognitive dual-task interventions in older adults. Only one study matching our search criteria examined the effects of simultaneous strength-cognition dual-task in older adults. Norouzi et al. (2019) indicated the positive impact of strength-based dual-task on working memory and balance performance when compared with strength-only or a control group. The same differences were maintained at 12 weeks follow-up. These results are promising because resistance training alone has been shown to improve processing speed and executive functions such as attention, inhibitory control, and mental flexibility, but not working memory (Li et al., 2018; Herold et al., 2019; Landrigan et al., 2020).

Instead, in a meta-analysis published by Bonnechère et al. (2020), cognitive training alone was found to benefit working memory, verbal memory, processing speed and executive function, but not visuospatial abilities or attention. Therefore, it is possible that simultaneous cognitive-resistance training impacts more dimensions of cognition when compared with only strength training or with only cognitive training (Norouzi et al., 2019). However, it should be noted that there are studies that observed no beneficial effects of adding further simultaneous cognitive training to multicomponent physical exercise training. For example, Rezola-Pardo et al. (2019) found no difference in the effectiveness of a 3-months strength-balance or strength-balance with simultaneous cognitive components on dual-task performance, physical performance, frailty scores, and loneliness perception in a sample of healthy older adults. The interventions did not affect executive function either. Only the strength-balance intervention had significant beneficial effects on the 6-min walking test, timed up and go test, anxiety, depression, and quality of life. Overall, they concluded that additional simultaneous cognitive training has no additional effects when compared with multicomponent exercise programs, because, the additional cognitive training may decrease or modify the intensity (e.g., velocity and power) of performing exercise training (Rezola-Pardo et al., 2019). This study was not included in the present systematic review, because of the multicomponent exercise regimen used where the effects of combined strength and cognitive training could not be separated.

The available literature included for the present systematic review supports these findings. Adding a further cognitive component to strength-based intervention can have a positive influence (Norouzi et al., 2019; Gutiérrez-Cruz et al., 2020), but with a suboptimal combination it can also impair the beneficial effects of strength training (Fiatarone Singh et al., 2014). Future studies regarding combined strength and cognitive training should ensure that the intended strength training exposure can be monitored and that it is not compromised by a too difficult or distracting cognitive or exergame part.

### Approaching to “Strength Exergaming”

Exergaming increases motivation for exercising (Skjæret et al., 2016; Stojan and Voelcker-Rehage, 2019; Gallou-Guyot et al., 2020) and can effectively improve health outcomes, especially in older adults (Di Lorito et al., 2021). However, further studies with rigorous designs are needed to draw firm conclusions. In their review, Stojan and Voelcker-Rehage concluded that exergaming appears to be more effective than sole physical exercise intervention on cognitive function in older adults, but further higher quality studies with better designed exergame components are required to yield more distinct results. Gallou-Guyot et al. (2020) discussed the available evidence and concluded that even though exergaming can have a positive impact on cognition, it does not seem to impact dual-task performance and the effects on physical functions remain controversial. Furthermore, the safety, feasibility, transfer effect, adherence and retention of benefits for both exergaming and dual-task intervention types are unclear and further studies are required to improve the quality of evidence (Gallou-Guyot et al., 2020). Various exergames (Dance Revolution, Xbox Kinect, Wii, Nintendo, OTAGO/FaME, and Mat) have been introduced and examined in interventional studies (Miyachi et al., 2010; Lyons et al., 2011; O'Donovan et al., 2012; Taylor et al., 2012; Stanmore et al., 2019; Peng et al., 2020). Overall, the existing exergames can be broadly categorized into three groups as follows: (1) commercial home video game consoles; (2) dance and step video games and, (3) interactive virtual ergometers (like a virtual kayak and cycle ergometer) (Stojan and Voelcker-Rehage, 2019). It seems that each category has varying effects on the brain and cognition, possibly due to different cognitive and physical efforts required (Stojan and Voelcker-Rehage, 2019). However, it is difficult to control the exergame intensity (Stanmore et al., 2017; Stojan and Voelcker-Rehage, 2019), which makes aerobic and resistance exercise training comparison difficult with regards to their effects on cognitive and functional outcomes (Bacha et al., 2018; Guimarães et al., 2018). Another important consideration is that although containing various physical exercise components (i.e., aerobic, strength and motor), the contemporary exergames do not include components of strength training corresponding to the current physical activity guidelines, therefore providing an insufficient stimulus to improve strength and functionality.

To the best of our knowledge, there is no evidence of the effectiveness of a strength-cognitive based exergame in the literature. However, several strength exergame concepts have been proposed and evaluated either from an entertainment or game programming perspective (Wang et al., 2018; Dash et al., 2019; Garcia-Hernandez et al., 2019; Lee et al., 2019; Lai et al., 2020). Considering the numerous health benefits of strength training, and that combining strength training with a simultaneous video game based cognitive part can further amplify the health and cognitive benefits and increase motivation, the development of new strength exergames is an attractive innovation possibility for companies and for researchers. The innovations need to be based on the evidence and consider the most potent mechanisms of how the given exposure of strength and cognitive training affects the outcomes. Although the current literature on the topic is only emerging and the available evidence is mostly of moderate quality, we propose that the potential advantages of such innovations are high, and the risks are low, so advancing the field further is well-justified. To support these endeavors, we suggest future studies should investigate how exergames should be designed specifically for the strength training regimen.

**Figure 2:**
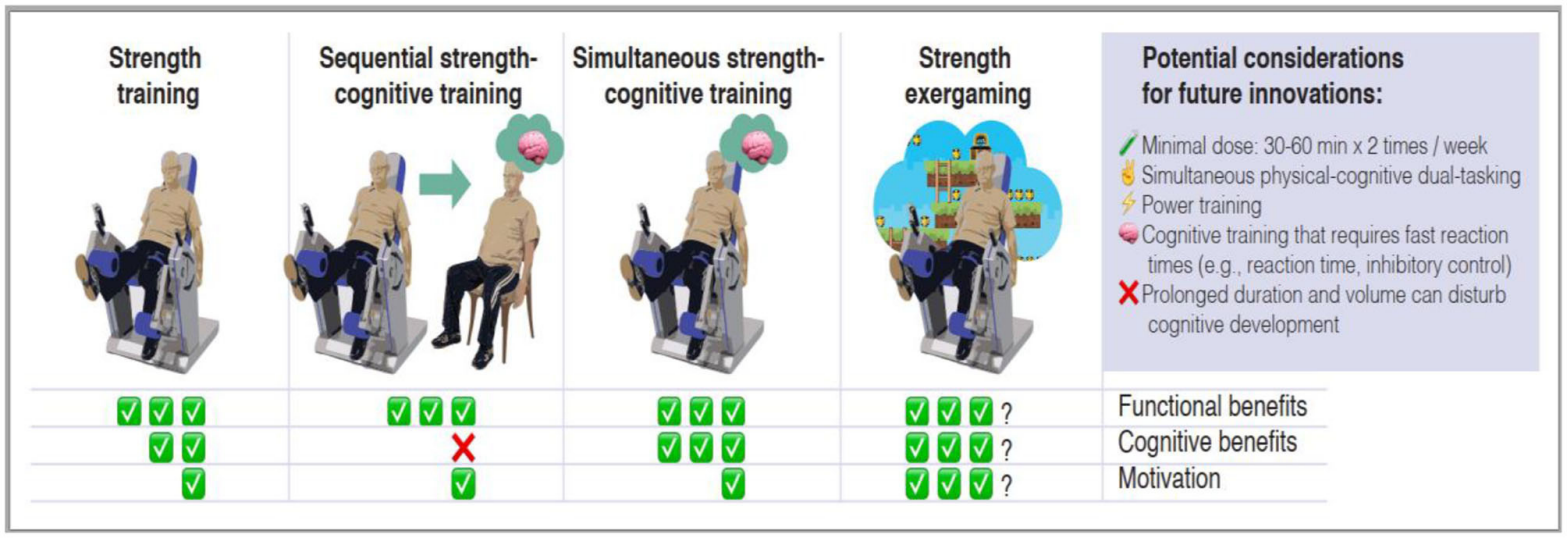
A suggested conceptual research framework for developing strength-cognitive exergames to increase strength training adherence and motivation in adults and the older adults.

In Figure 2, we propose the established and plausible benefits, as well as the key considerations for a strength-cognition exergame to be effective. Strength training is one of the most beneficial exercise interventions, especially for older adults given the numerous health, functional, psychological and cognitive benefits it provides (Fisher et al., 2017; Papa et al., 2017; Fragala et al., 2019; McLeod et al., 2019; Di Lorito et al., 2021). On the other hand, simultaneous physical-cognitive dual-task training has been reported to be more effective than sequent physical and cognitive training (Tait et al., 2017); and the key benefits of exergaming include improved cognitive and functional outcomes in older adults (Di Lorito et al., 2021). Furthermore, exergaming-based interventions are appealing and interesting for older adults and therefore can assist older adults in leading an active aging processes (Vázquez et al., 2018). Finally, cognitive based exergames are more effective than cognitive-motor dual-task exercises on brain and cognition in older adults (Bruderer-Hofstetter et al., 2018; Lord and Close, 2018). Consequently, by combining strength training with cognitive video games, it is possible to promote a novel “strength exergaming” to increase not only motivation but also possibly effectiveness on cognition and function in adults and older adults if the basic principles of strength training are followed (Figure 2). The minimal dose for physiological, psychological, and functional health benefits of strength training has been reported to be at least 30–60 min in each session, performed at least twice per week (Fisher et al., 2017; Fragala et al., 2019). However, due to non-linear association between different types of resistance exercise (e.g., hypertrophic or power training) and their effectiveness on cognition, the intensity at each session should be considered according to the type of resistance exercise (Daniel Gallardo-Gomez et al., 2022). To effectively impact cognitive function in older adults, training with resistance exercise cluster, resistance bands and body-and-free weights and machines in older adults should be done in volumes between 474 and 77 METs-min/week, 78–679 METs-min/week and 529–891 METs-min/week, respectively (Huang et al., 2021; Daniel Gallardo-Gomez et al., 2022). In addition to the volume, increasing the speed of repetitions, or power, can further increase effectiveness (Sayers and Gibson, 2014; Cadore and Izquierdo, 2018). From the daily functionality point of view power training can be especially beneficial (Hazell et al., 2007). In addition, power training impacts cognition similarly (Cherup et al., 2018; Coelho-Júnior et al., 2020), but is more beneficial for physical function, compared to strength training alone (Hazell et al., 2007). Therefore, cognitive training that requires fast reaction times (e.g., reaction time, inhibitory control) can be particularly effective.

In order to maximize safety and to lower the participation barrier, the equipment/methods should be simple and accessible. For example, machine-based devices enabling single joint movements and exercise volume and intensity control can be optimal for older adults and novices (Fisher et al., 2017; Fragala et al., 2019; Netz, 2019). In addition, such simple and uncomplicated devices would make it possible to perform the desired intensity and volume of strength training simultaneously with video game based cognitive tasks (Angevaren et al., 2007; Stanmore et al., 2017; Netz, 2019; Stojan and Voelcker-Rehage, 2019), without allowing the cognitive component to interfere with strength training which is a confounding factor in the literature (Fiatarone Singh et al., 2014; Stanmore et al., 2017; Joubert and Chainay, 2018; Rezola-Pardo et al., 2019; Stojan and Voelcker-Rehage, 2019). Furthermore, strength training alone has not been effective in influencing working memory (Landrigan et al., 2020), but combining it with simultaneous cognitive tasks (Eckardt and Rosenblatt, 2019) and memory tasks (Norouzi et al., 2019) can impact positively working memory. Therefore, also the specificity of the cognitive component should be considered along with the type of strength exposure, lack of interference, and other key factors maximizing the effectiveness of strength exergaming (Figure 2). Finally, strength training programs should be designed to include motivational components (such as programs built upon self-determination theory) to increase autonomy in performing strength training. Motivationally enriched programs have been shown impact positively physical, psychological, and social levels in older adults (Marcos-Pardo et al., 2018; Martínez-Rodríguez et al., 2021). Considering the proposed strength-exergame concept, such future innovations should consider incorporating such motivational components within the strength-exergame game design.

## Conclusion

Strength training is one of the most potential interventions to improve health and functionality and to prevent cognitive decline of adults. The barriers for participation include lack of motivation, resulting in low adherence and the decreased effectiveness of strength training. On the other hand, various exergames have been introduced that can increase motivation and adherence to aerobic and multicomponent training regimens. We found little information on the effectiveness of strength exergames; that is, exergames, like video games, that would have combined strength training specifically at the recommended doses. However, we found some evidence of the effectiveness of combined sequent or combined strength and cognitive training. While cognitive training does not include a gamified component and therefore is a different concept to exergaming, some of the mechanisms, such as dual-tasking, are shared. Two of the three studies reported beneficial effects of adding a further cognitive component to strength training on cognition and function, but the study that examined the addition of a sequent cognitive component to strength training showed negative effects on cognition. Due to the scant evidence, it is not possible to draw comprehensive conclusions. As such, there is a need for more randomized controlled trials (RCT) studies with various methodologies, such as including various experimental groups such as strength, cognitive, dual-task and dual-task-strength in the same study as well including both active and passive control groups, and preventing the cognitive component interfering with the strength exercise. Despite the low number of evidence available currently, the potential benefits of strength training or exergaming are enormous. Researchers and companies are encouraged to combine simultaneous strength and cognitive training to innovate new strength exergames that can engage adults and older adults in effective strength and cognitive training to further improve their quality of life and physical mobility. The following suggestions can be considered: (1) ensure minimal dosage of strength training (30–60 min, 2 × /week), (2) use machine-based strength training devices to control volume and intensity (to prevent cognitive components from interfering with strength training), (3) include power training by using cognitive tasks requiring rapid reactions, and (4) add cognitive memory tasks (to extend the cognitive benefits of strength training *per se*), and (5) include motivational components to increase training adherence.

## Data Availability Statement

The raw data supporting the conclusions of this article will be made available by the authors, without undue reservation.

## Author Contributions

SE and AP planned the systematic review. SE and SK conducted the search. AP and SK undertook the senior review of the work on completion of the manuscript preparation. SE is responsible for the overall content of the study. All authors contributed equally to the writing of the paper.

## Conflict of Interest

The authors declare that the research was conducted in the absence of any commercial or financial relationships that could be construed as a potential conflict of interest.

## Publisher's Note

All claims expressed in this article are solely those of the authors and do not necessarily represent those of their affiliated organizations, or those of the publisher, the editors and the reviewers. Any product that may be evaluated in this article, or claim that may be made by its manufacturer, is not guaranteed or endorsed by the publisher.
